# Traveling wave solution and qualitative behavior of fractional stochastic Kraenkel–Manna–Merle equation in ferromagnetic materials

**DOI:** 10.1038/s41598-024-63714-4

**Published:** 2024-06-06

**Authors:** Jie Luo

**Affiliations:** School of Electronic Information and Electrical Engineering, Chengdu University, Chengdu, 610106 People’s Republic of China

**Keywords:** Kraenkel–Manna–Merle equations, Bifurcation, Phase portrait, Plane dynamics system, Nonlinear phenomena, Information theory and computation

## Abstract

The main purpose of this article is to investigate the qualitative behavior and traveling wave solutions of the fractional stochastic Kraenkel–Manna–Merle equations, which is commonly used to simulate the zero conductivity nonlinear propagation behavior of short waves in saturated ferromagnetic materials. Firstly, fractional stochastic Kraenkel–Manna–Merle equations are transformed into ordinary differential equations by using the traveling wave transformation. Secondly, the phase portraits, sensitivity analysis, and Poincaré sections of the two-dimensional dynamic system and its perturbation system of ordinary differential equations are drawn. Finally, the traveling wave solutions of fractional stochastic Kraenkel–Manna–Merle equations are obtained based on the analysis theory of planar dynamical system. Moreover, the obtained three-dimensional graphs of random solutions, two-dimensional graphs of random solutions, and three-dimensional graphs of deterministic solutions are drawn.

## Introduction

In recent years, fractional stochastic partial differential equations^1–3^ have been commonly used to simulate nonlinear problems affected by random factors in fields of physics, chemistry, biology, and engineering technology. Especially in the modeling process of complex system^4–6^, the influence of random factor is inevitable. Because considering random factor is more in line with the actual situation in these natural science fields. In recent years, many stochastic fractional order partial differential equations have been studied. For example, fractional stochastic Schrödinger equations^7^, stochastic fractional fokas system^8^, stochastic fractional Hirota–Maccari system^9^, fractional stochastic Broer–Kaup equations^10^, stochastic fractional RKL equation^11^, fractional stochastic potential Yu–Toda–Sasa–Fukuyama equation^12^, fractional-stochastic quantum Zakharov–Kuznetsov equation^13^, stochastic-fractional Drinfel’d–Sokolov–Wilson equations^14^, etc. In the study of the above equations, the most important task is to construct accurate traveling wave solutions^15–29^, and many effective methods have been proposed to construct traveling wave solutions for fractional stochastic partial differential equations.

In this study, the fractional stochastic Kraenkel–Manna–Merle equations are presented as follows^30^

1.1 $$\begin{aligned} {\left\{ \begin{array}{ll} \mathfrak {D}_{x}^{\alpha }\varphi _{t}-\varphi \mathfrak {D}_{x}^{\alpha }\psi +\kappa \mathfrak {D}_{x}^{\alpha }\psi =\sigma \mathfrak {D}_{x}^{\alpha }\varphi \mathfrak {B}_{t},\\ \mathfrak {D}_{x}^{\alpha }\psi _{t}-\varphi \mathfrak {D}_{x}^{\alpha }\varphi =\sigma \mathfrak {D}_{x}^{\alpha }\psi \mathfrak {B}_{t}, \end{array}\right. } \end{aligned}$$

where the magnetization and the external magnetic field related to the ferrite are represented by $\varphi =\varphi (t,x)$ and $\psi =\psi (t,x)$, respectively. $\mathfrak {D}_{x}^{\alpha }$ stands for the conformable fractional derivative. $\kappa$ is the coefficient of the damping. $\sigma$ represents the noise intensity. $\mathfrak {B}$ stands for the Brownian motion, and $\mathfrak {B}_{t}=\frac{\partial \mathfrak {B}}{\partial t}$. In^30^, Wael W. Mohammed et al. constructed the traveling wave solution of Eq. (1.1) by the Mapping method. The main purpose of this article is to study the phase diagram and traveling wave solutions of Eq. (1.1) by the theory of dynamical systems. At the same time, by adding small perturbations to the two-dimensional dynamical system, its chaotic behavior, sensitivity, and Poincaré sections are considered.

The remaining sections in this article are arranged as follows: In Sect. 2, the phase portraits of the dynamical system and its perturbed system are discussed. In Sect. 3, the solutions of Eq. (1.1) are constructed by the method of analyzing planar dynamical systems. Finally, a brief conclusion is given.

## Bifurcation and chaotic behaviors

### Preliminary

#### Definition 2.1

^31^ For $\alpha \in (0,1]$, the conformable fractional derivative of $f:\mathbb {R}^{+}\rightarrow \mathbb {R}$ is defined as

$$\begin{aligned} \mathfrak {D}_{t}^{\alpha }f(t)=\lim _{h\rightarrow 0}\frac{f(t+ht^{1-\alpha })-f(t)}{h}. \end{aligned}$$

#### Definition 2.2

^32^ The Brownian motion $\{\mathfrak {B}(t)\}_{t\ge 0}$ is a stochastic process and satisfies:

(i) $$\mathfrak {B}(0)=0$$;
(ii) $\mathfrak {B}(t)$ is continuous for $t\ge 0$;
(iii) $\mathfrak {B}(t_{2})-\mathfrak {B}(t_{1})$ is independent for $t_{2}>t_{1}$;
(iv) $\mathfrak {B}(t_{2})-\mathfrak {B}(t_{1})$ has a normal distribution $N(0,t_{2}-t_{1})$.

#### Lemma 2.3

^33^ $\mathbb {E}(e^{\rho \mathfrak {B}(t)})=e^{\frac{1}{2}\rho ^{2}t}$ for $\rho \ge 0$, where $\mathbb {E}$ represents mathematical expectation.

### Mathematical derivation

When $\kappa =0$, the wave transformation is considered

2.1 $$\begin{aligned} \begin{aligned} \varphi (t,x)=\Phi (\xi )\textbf{e}^{\sigma \mathfrak {B}(t)-\frac{1}{2}\sigma ^{2}t},\quad \psi (t,x)=\Psi (\xi )\textbf{e}^{\sigma \mathfrak {B}(t)-\frac{1}{2}\sigma ^{2}t},\quad \xi =\frac{1}{\alpha }k_{1}x^{\alpha }+k_{2}t, \end{aligned} \end{aligned}$$

here $\Phi (\xi )$ and $\Psi (\xi )$ are real functions. $k_{1}$ and $k_{2}$ stand for nonzero constants.

Inserting Eq. (2.1) into Eq. (1.1), we have

2.2 $$\begin{aligned} {\left\{ \begin{array}{ll} k_{1}k_{2}\Phi ^{''}-k_{1}\Phi \Psi '\textbf{e}^{\sigma \mathfrak {B}(t)-\frac{1}{2}\sigma ^{2}t}=0,\\ k_{1}k_{2}\Psi ^{''}-k_{1}\Phi \Phi '\textbf{e}^{\sigma \mathfrak {B}(t)-\frac{1}{2}\sigma ^{2}t}=0. \end{array}\right. } \end{aligned}$$

By taking the expected values on both sides of Eq. (2.2) and using Lemma 2.3, it can be concluded that

2.3 $$\begin{aligned} {\left\{ \begin{array}{ll} k_{2}\Phi ^{''}-\Phi \Psi '=0,\\ k_{2}\Psi ^{''}-\Phi \Phi '=0. \end{array}\right. } \end{aligned}$$

Integrating both sides of the second equation of Eq. (2.3) simultaneously yields

2.4 $$\begin{aligned} \Psi '=\frac{1}{2k_{2}}\Phi ^{2}+\frac{c_{0}}{k_{2}}, \end{aligned}$$

where $c_{0}$ is the integration constant.

Inserting Eq. (2.4) into the first equation of Eq. (2.3) yields

2.5 $$\begin{aligned} \Phi ^{''}-\varpi _{1}\Phi ^{3}-\varpi _{2}\Phi =0, \end{aligned}$$

where $$\varpi _{1}=\frac{1}{2k_{2}^{2}}$$ and $$\varpi _{2}=\frac{c_{0}}{k_{2}^{2}}$$.

### Phase portraits

The two-dimensional dynamics of system (2.5) are described as follows

2.6 $$\begin{aligned} {\left\{ \begin{array}{ll} \frac{d\Phi }{d\xi }=z,\\ \frac{dz}{d\xi }=\varpi _{1}\Phi ^{3}+\varpi _{2}\Phi , \end{array}\right. } \end{aligned}$$

with Hamiltonian function

2.7 $$\begin{aligned} H(\Phi ,z)=\frac{1}{2}z^{2}-\frac{\varpi _{1}}{4}\Phi ^{4}-\frac{\varpi _{2}}{2}\Phi ^{2}=h, \end{aligned}$$

where *h* is the Hamiltonian constant.

Assume that the root of $F(\Phi _{j})=0$ ($j=0,1,2$) is the abscissa of the equilibrium point, where $F(\Phi _{j})=\varpi _{1}\Phi _{j}^{3}+\varpi _{2}\Phi _{j}$. Suppose that $$\textbf{M}(\Phi _{j},0)=\left( \begin{matrix} 0 &{} 1\\ 3\varpi _{1}\Phi _{j}^{2}+\varpi _{2}&{} 0 \end{matrix}\right) $$ is the coefficient matrix of (2.6) at the equilibrium point. Then, we obtain

2.8 $$\begin{aligned} \textbf{det}(\textbf{E}(\Phi _{j},0))=-F'(\Phi _{j}),\ \ j=0,1,2. \end{aligned}$$

#### Remark 2.4

**Figure 1:**
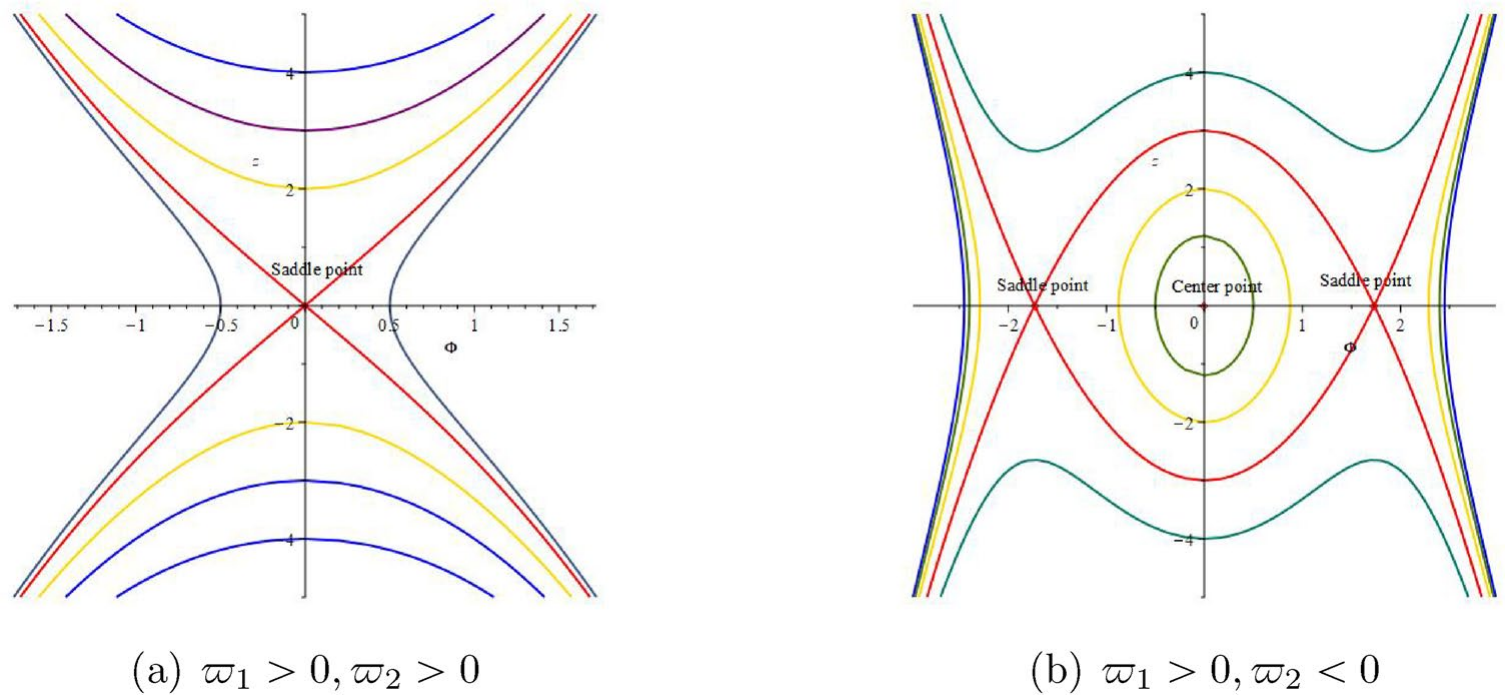
2D phase portrait of system (2.6).

When $\varpi _{1}>0$ and $\varpi _{2}>0$, the system (2.6) has one equilibrium point (0, 0), which is a saddle point as shown in Fig. 1a. When $\varpi _{1}>0$ and $\varpi _{2}<0$, the system (2.6) has three equilibrium point (0, 0), $(\sqrt{-\frac{\varpi _{2}}{\varpi _{1}}},0)$, $(-\sqrt{-\frac{\varpi _{2}}{\varpi _{1}}},0)$, (0, 0) is the center point as shown in Fig. 1b. $(\sqrt{-\frac{\varpi _{2}}{\varpi _{1}}},0)$ and $(-\sqrt{-\frac{\varpi _{2}}{\varpi _{1}}},0)$ are the saddle points by using Maple 2022 mathematical software as shown in Fig. 1b.

### Chaotic behaviors

Therefore, system (2.6) can be transformed into the following two-dimensional disturbance system with perturbation term

2.9 $$\begin{aligned} {\left\{ \begin{array}{ll} \frac{d\Phi }{d\xi }=z,\\ \frac{dz}{d\xi }=\varpi _{1}\Phi ^{3}+\varpi _{2}\Phi +f(\xi ), \end{array}\right. } \end{aligned}$$

where $f(\xi )=A\cos (k\xi )$ and $f(\xi )=Ae^{-0.05\xi }$ are the perturbed term. *A* represent the amplitude of system (2.9). *k* stands for the frequency of system (2.9).

#### Remark 2.5

**Figure 2:**
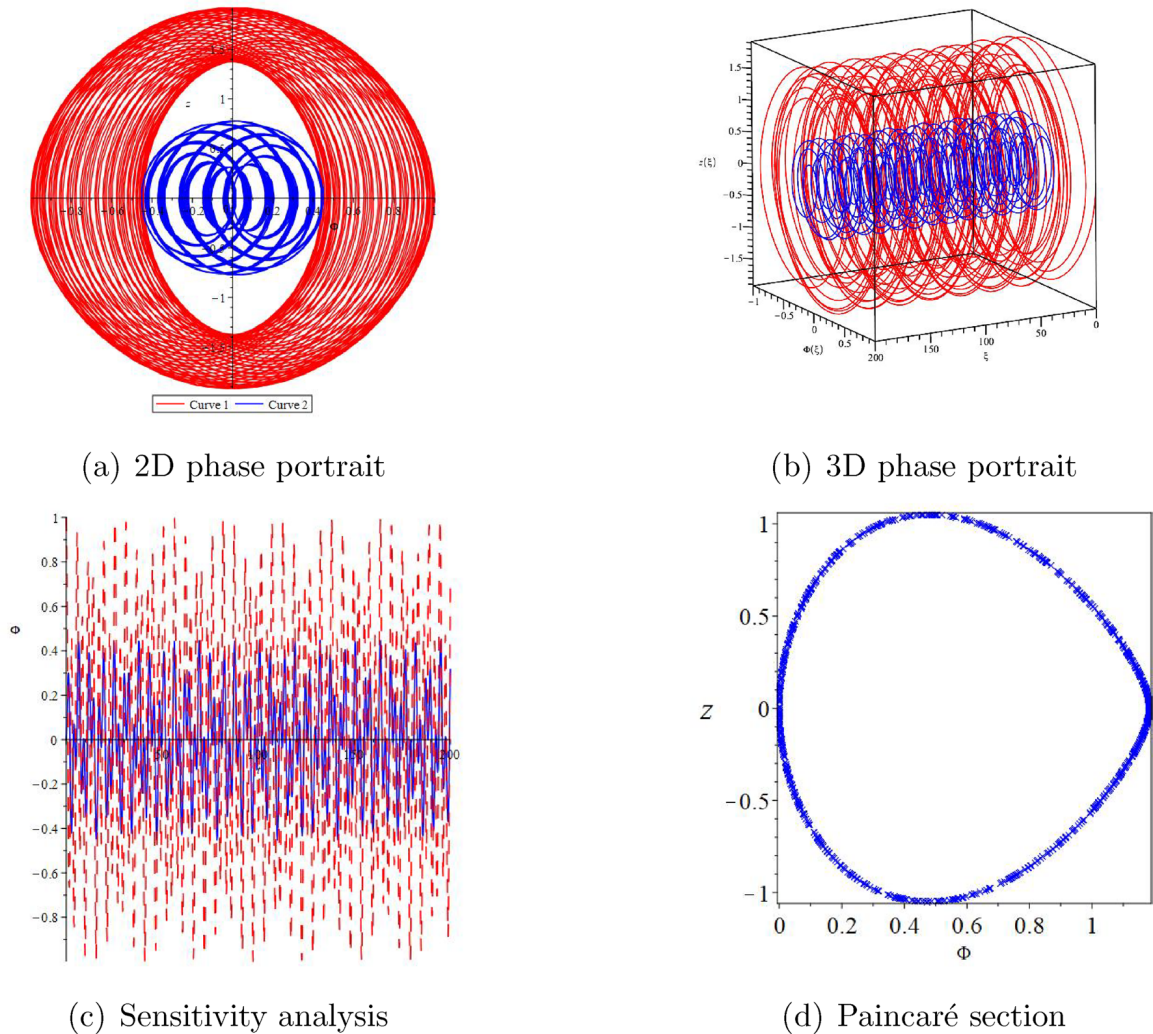
The chaotic behaviors of system (2.9) with $\varpi _{1}=2,\varpi _{2}=-6,A=2.9,k=1$.

**Figure 3:**
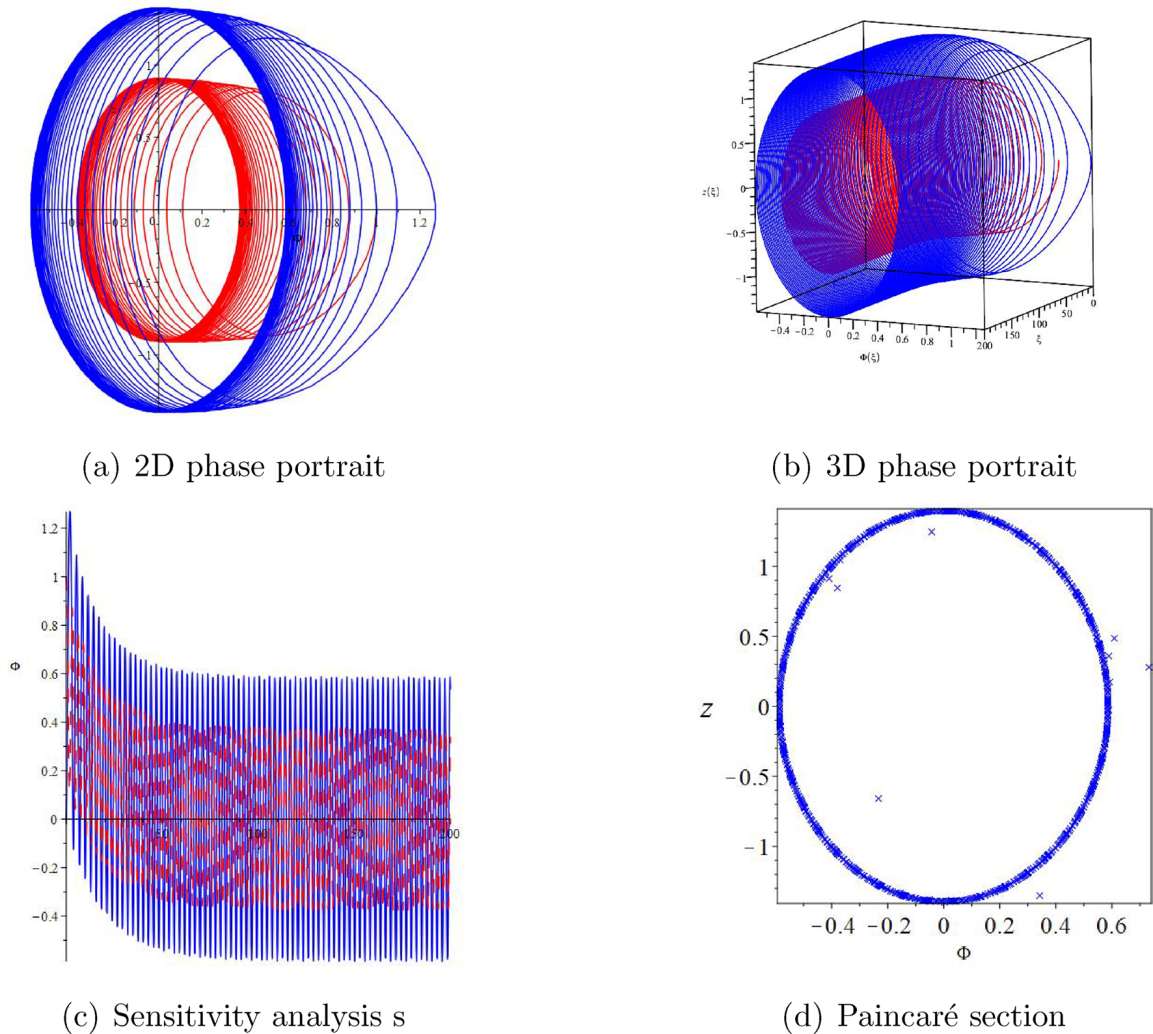
The chaotic behaviors of system (2.9) with $\varpi _{1}=2,\varpi _{2}=-6,A=2.9$.

Here, we have used the Maple 2022 mathematical software to draw two-dimensional diagrams, three-dimensional diagrams, sensitivity analysis, and Paincaré section of the disturbance system (2.9) (see Figs. 2 and 3). The disturbance systems we are considering are periodic disturbances and small disturbance systems. At the same time, we also considered the 2D phase diagram, 3D phase diagram, and sensitivity analysis of the disturbance system (2.9) under different initial values.

## Traveling wave solution of Eq. (1.1)

Firstly, we make some assumptions $h_{0}=H(0,0)=0$, $h_{1}=H(\pm \sqrt{-\frac{\varpi _{1}}{\varpi _{2}}},0)=\frac{\varpi _{2}^{2}}{4\varpi _{1}}$.

### $\varpi _{1}>0$, $\varpi _{2}<0$, $0<h<\frac{\varpi _{2}^{2}}{4\varpi _{1}}$

Then system (2.6) becomes

3.1 $$\begin{aligned} z^{2}=\frac{\varpi _{1}}{2}(\Phi ^{4}+\frac{2\varpi _{2}}{\varpi _{1}}\Phi ^{2}+\frac{4h}{\varpi _{1}}) =\frac{\varpi _{1}}{2}(\kappa _{1h}^{2}-\Phi ^{2})(\kappa _{2h}^{2}-\Phi ^{2}), \end{aligned}$$

where $$\kappa _{1h}^{2}=\frac{-\varpi _{2}+\sqrt{\varpi _{2}^{2}-4\varpi _{1}h}}{\varpi _{1}}$$ and $$\kappa _{2h}^{2}=\frac{-\varpi _{2}-\sqrt{\varpi _{2}^{2}-4\varpi _{1}h}}{\varpi _{1}}$$.

Substituting (3.1) into $\frac{d\Phi }{d\xi }=z$ and integrating it, we can present the following integral equation

3.2 $$\begin{aligned} \int ^{\Phi }_{0}\frac{d\Xi }{\sqrt{(\kappa _{1h}^{2}-\Xi ^{2})(\kappa _{2h}^{2}-\Xi ^{2})}}=\mp \sqrt{\frac{\varpi _{1}}{2}}(\xi -\xi _{0}), \end{aligned}$$

From Eq. (3.2), the Jacobian function solutions can be presented

3.3 $$\begin{aligned} \begin{aligned} \varphi _{1}(t,x)=\pm \kappa _{1h}\textbf{sn}(\kappa _{2h}\sqrt{\frac{\varpi _{1}}{2}}(\frac{1}{\alpha }k_{1}x^{\alpha }+k_{2}t),\frac{\kappa _{1h}}{\kappa _{2h}})\textbf{e}^{\sigma \mathfrak {B}(t)-\frac{1}{2}\sigma ^{2}t}. \end{aligned} \end{aligned}$$

### $\varpi _{1}>0$, $\varpi _{2}<0$, $h=\frac{\varpi _{2}^{2}}{4\varpi _{1}}$

Then, we obtain $\kappa _{1h}^{2}=\kappa _{2h}^{2}=-\frac{\varpi _{2}}{\varpi _{1}}$. Similar to situation 3.1, we can obtain through integration

3.4 $$\begin{aligned} \begin{aligned} \varphi _{2}(t,x)=\pm \sqrt{-\frac{\varpi _{2}}{\varpi _{1}}}\textbf{tanh}(\sqrt{-\frac{\varpi _{2}}{2}}(\frac{1}{\alpha }k_{1}x^{\alpha }+k_{2}t))\textbf{e}^{\sigma \mathfrak {B}(t)-\frac{1}{2}\sigma ^{2}t}. \end{aligned} \end{aligned}$$

#### Remark 3.1

Through the transformation (2.1) and relationship (2.4), we can easily obtain another solution $\psi _{1}(t,x)$ and $\psi _{2}(t,x)$ to Eq. (1.1) by using Eqs. (3.3) and (3.4).

### Numerical simulations

**Figure 4:**
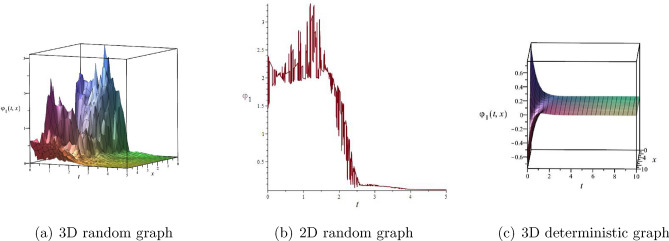
The solution $\varphi _{1}(t,x)$ with $k_{1}=\frac{\sqrt{2}}{2},k_{2}=\frac{\sqrt{2}}{2},c_{0}=-1,\varpi _{1}=1,\varpi _{2}=-2,\sigma =\frac{1}{2},\alpha =\frac{1}{2},h=\frac{1}{2}$.

**Figure 5:**
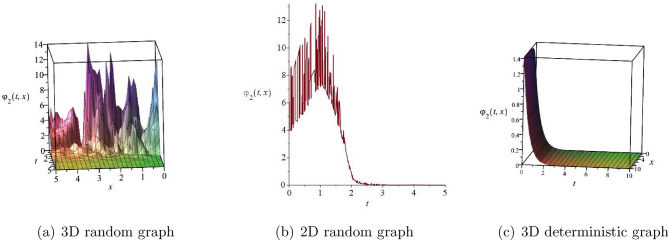
The solution $\varphi _{2}(t,x)$ with $k_{1}=\frac{\sqrt{2}}{2},k_{2}=\frac{\sqrt{2}}{2},c_{0}=-1,\varpi _{1}=1,\varpi _{2}=-2,\sigma =\frac{1}{2},\alpha =\frac{1}{2},h=1$.

In this section, we plotted the solutions $\psi _{1}(t,x)$ and $\psi _{2}(t,x)$ including three-dimensional random graphs, two-dimensional random graphs, and three-dimensional deterministic graphs by setting different parameters and Maple 2022 mathematical software as shown in Figs. 4 and 5. $\psi _{1}(t,x)$ is a Jacobian function solution. $\psi _{2}(t,x)$ is a bell shaped solitary wave solution.

## Conclusion

This article uses the theory of dynamical systems to study the traveling wave solutions and qualitative behavior of Eq. (1.1). Compared with reference^30^, this article not only obtained the traveling wave solution of Eq. (1.1), but also analysed the chaotic behavior, sensitivity analysis, and Poincaré sections by adding small perturbations. In this article, we consider two different forms of disturbance systems. On the one hand, we consider periodic perturbation system. On the other hand, we also considered the dynamic behavior of small periodic disturbances. In order to facilitate readers’ understanding of the solution of Eq. (1.1), we have drawn three-dimensional and two-dimensional graphs of the solutions $\psi _{1}(t,x)$ and $\psi _{2}(t,x)$, as well as three-dimensional graphs without considering the influence of random factors. In future research, our focus will be on the study of traveling wave solutions and dynamic behavior of more complex fractional order stochastic partial differential equations.

## Data Availability

The datasets used and/or analyzed during the current study available from the corresponding author on reasonable request.
